# The protective effects of melatonin on testis, sperm parameters quality, and in-vitro fertilization in mice following treatment with aflatoxin B1: An experimental study

**DOI:** 10.18502/ijrm.v23i2.18492

**Published:** 2025-05-01

**Authors:** Maryam Sabahi, Mojtaba Karimipour, Abass Ahmadi, Bagher Pourheydar, Gholamhossein Farjah

**Affiliations:** ^1^Department of Anatomical Sciences, School of Medicine, Urmia University of Medical Sciences, Urmia, Iran.; ^2^Laboratory of Embryology, Department of Basic Sciences, Faculty of Veterinary Medicine, Urmia University, Urmia, Iran.

**Keywords:** Melatonin, Aflatoxin B1, Sperm, Fertilization, Mice.

## Abstract

**Background:**

Aflatoxin B1 (AFB1) contamination of foods and animal feeds is a public health issue. Exposure to AFB1 induces oxidative stress and can cause male reproductive toxicity. Melatonin (MLT) is a neuro-hormone produced by the pineal gland and the testis and is known as a potent antioxidant.

**Objective:**

This study aims to determine the protective effect of MLT on testicular tissue alterations, sperm parameter indexes, and in vitro fertility assays in mice treated with AFB1.

**Materials and Methods:**

In this experimental study, 28 adult male NMRI mice (8–10 wk old, 25–27 gr) were divided randomly into 4 groups: control, MLT (20 mg/kg/day, intraperitoneally), AFB1 (50 μ g/kg/day, intraperitoneally) and MLT+AFB1. After 35 consecutive days, testis histological changes, sperm quality parameters, the rate of sperm with DNA damage, and in vitro fertilization outcomes up to the blastocyst stage were surveyed and compared between groups.

**Results:**

Our results showed that AFB1 administration induced histological alterations in the testis and signiﬁcantly decreased all the sperm parameters and in vitro fertility (fertilization and blastocyst formation rates) compared to control. Additionally, the percentages of immature sperms and sperms with DNA damage significantly (p < 0.001) increased in the AFB1-treated group. MLT treatment in the MLT+AFB1group significantly increased testis quality and sperm parameters and improved in vitro fertilizaton rate and in vitro embryonic development.

**Conclusion:**

These findings demonstrated that MLT can compensate for the adverse effects of AFB1 on the quality of testicular tissue, sperm parameters, sperm DNA, and in vitro fertilization outcomes.

## 1. Introduction

Globally, infertility is an important medical problem and has been estimated in 48.5 million couples, having negative impacts on various aspects such as psychological, social, personal, and economic (1). There are many major risk factors that affect male fertility. Among these, aflatoxins (AF) are well known as the most highly toxic agent that induces reproductive toxicity including, testicular damage, and decreases sperm quality in humans and various animal species (2, 3). The reproductive system is the vulnerable target of Aflatoxin B1 (AFB1), and it has been well documented that AFB1 negatively influences both male and female reproductive function. Recently, it has been shown that AFB1 exposure during in vitro porcine oocyte maturation significantly disrupted nuclear and cytoplasmic maturation (4). A growing number of research have focused on the adverse effects of AFB1 on male reproductive function. Some studies confirmed that AFB1, through suppressing the antioxidant defense system, induces oxidative stress injury in testicular tissue and disrupts normal spermatogenesis (5). In a recent study, it was reported that AFB1 exposure in mice leads to a significant reduction in sperm quality parameters and also significantly increased sperms with morphological abnormality (6). Moreover, exposure to AFB1 not only induces testicular tissue damage and reduces sperm parameters quality but can also disrupt testosterone production from Leydig cells, which plays a main role in spermatogenesis (7, 8). Several studies confirmed that AFB1-induced organ damage is related to oxidative stress, inflammation, and apoptosis (9–11).

Considering that exposure to AF is unavoidable and is also considered as serious health problem thus, it is important to find therapeutic or protective agents like antioxidants against AF-induced toxicity. In this light, a series of researchers used various chemical compounds to mitigate AFB1-induced damage to testis. For example, a study done in 2019 showed that resveratrol effectively prevents testicular damage and lipid peroxidation due to AF exposure in rats (12). In another study, the protective effects of selenium nanoparticles against AF-induced damaged testis and spermatogenesis in mice were reported (3).

Melatonin (MLT) is a small endogenous neurohormone mainly produced by the pineal gland and is well-defined as a potent free-radical scavenger. MLT is also synthesized by testis and regulates its function (13).

In previous studies, the protective effects of MLT on testis were repeatedly reported against oxidative stress, inflammation, and apoptosis. In an experimental study, it was elucidated that treatment with MLT reduced apoptosis and oxidative stress and also promoted recovery of the testis from heat-induced damage in mice (13). On the other hand, protective effects of MLT are reported in testis injuries induced by chemotherapy, diabetes, and nicotine (14–16).

Therefore, MLT might be useful as a potential agent to counteract the adverse effects of AFB1-induced testis damage, because of its advantages including high efficacy, free radical detoxification, and low toxicity. However, to the best of our knowledge there are no experimental studies that ascertain the protective effects of MLT against adverse effects of AFB1 on testis, sperm parameters quality, in vitro fertilization rate, and in vitro embryo development. Based on available findings, we hypothesized that MLT would be able to protect male reproductive function from injuries induced by AFB1 exposure. Thus, the current work aimed to investigate the potential protective effects of exogenous MLT on testis, spermatogenesis, in vitro fertilization rate, and in vitro embryonic development in adult mice treated with AFB1.

## 2. Materials and Methods

### Animals and treatment

In this experimental study, 28 adult male NMRI mice (8–10 wk old, 25–27 gr) were obtained from the Laboratory Animal Recourses Unit, Urmia University of Medical Sciences, Urmia, Iran. The animals were kept in standard environmental conditions at a temperature of 22 ± 2 C with a 12 hr light/dark cycle with free access to food and tap water throughout the study. The study was performed in the Anatomical Sciences Department of Urmia University of Medical Sciences, Urmia, Iran, from November 2020 to July 2021. After 1 wk of adaptation, the male mice were randomly divided into 4 groups (n = 7/each). The mice were treated once daily for 35 days as follows:

• Group 1, the control (Cont) group, was administrated with the vehicle.

• Group 2, the MLT group, daily dose of 20 mg/kg/ MLT (Sigma, USA) body weight (13).

• Group 3, AFB1 group, daily dose of 50 μ g/kg/ AFB1 (Sigma, USA) body weight (17).

• Group 4, AFB1 + MLT group was administrated with MLT (as in group 2) and AFB1 (as in group 3) every day.

AFB1 was dissolved in corn oil and ethanol (95:5, v/v), and all treatments were given intraperitoneally. At the end of the experiment the mice were sacrificed by cervical dislocation, and testicular histomorphometry, sperm parameters, and in vitro fertilization assay were analyzed.

### Testicular histomorphometry

The left testes of the animals were dissected and fixed in 10% formalin. After processing the tissue, 5 μ m sections with hematoxylin and eosin staining were prepared. Seminiferous tubule (SNTs) diameter, epithelium thickness, and spermiogenesis index (SPI) were determined. To measure SNT diameter, 10 round-shaped tubules were randomly selected, and the average of 2 diameters perpendicular to each other were calculated. To determine the epithelium thickness of these tubules, the average thickness at 2 locations from the basement membrane to the luminal surface was measured (18, 19). “To analyze the SPI in each mouse, 20 SNTs were randomly considered, and the percentage of SNTs with sperm in the lumen was calculated” (19).

### Sperm collection for the assessment of sperm parameters

To obtain sperm, the tail of the epididymides of each mouse were collected and minced “in pre-warmed dishes containing 1 mL human tubal fluid medium (HTF; Sigma, USA) supplemented with 4 mg/mL bovine serum albumin (BSA; Sigma, USA). Then, the dishes were incubated at 37 C in 5% CO_2_ to swim out the sperm into the medium. To assess sperm motility, 10 µL of each sperm sample was placed on a preheated Neubauer slide and then using a light microscope with × 400 magnification, the percentage of sperm with motility was calculated. In the current study, the sperms with no movement at all were considered non motile and the ones that displayed movements” (progressive motility + non progressive motility) were considered motile. To assess sperm density, a 1:50 dilution from sperm sample with water was prepared, and then using a Neubauer slide under a light microscope with × 400 magnification, sperm were counted. For evaluating sperm viability, 20 µL of sperm solution was mixed with the same volume of eosin and nigrosine stain and smears were prepared. Then, following the drying of the slides at room temperature, the percentage of red-colored dead sperms and alive unstained ones with no color were calculated. Finally, to assess the rate of sperms with abnormal morphology, the smears were obtained and stained with aniline blue (19).

### Sperm DNA assessment

Acridine orange staining was performed to detect sperm DNA damage and “reflects sperm chromatin denaturation (single-stranded DNA vs. double-stranded DNA). Briefly, the sperm smear slides from each” sample were obtained and then fixes in Carnoy's fixative (methanol/acetic acid 3:1). After drying at room temperature, the slides were stained by acridine orange for 7 min. Finally, using a fluorescent microscope, the percentage of normal sperms with green color and abnormal ones with yellow to red color was calculated (19).

### Sperm nucleus quality assessment

“Aniline blue staining was used to evaluate sperm nucleus maturity. In short, the dried smear slides were placed in 3% glutaraldehyde and then stained with 5% aniline blue for 7 min. At least using a light microscope, 200 sperms in each slide were counted, and the percentage of abnormal, immature sperm with dark blue color was determined” (19).

### Oocytes collection and in vitro fertilization (IVF) analysis

55 healthy adult female mice (8–10 wk old) were used for IVF assay. To collect mature oocytes, the female mice were superovulated by the injection of 10 IU of the pregnant mare's serum gonadotropin hormone (Intervet, Boxmeer, Netherlands) followed by 10 IU of human chorionic gonadotropin (hCG) after 48 hr. The mice were euthanized 12–14 hr after hCG injection, and the oviducts were collected and placed in human tubal fluid medium supplemented with 4 mg/mL bovine serum albumin, which had already preheated in an incubator at 37 C in 5% CO_2_. Then, the oocytes were obtained from oviducts and, after washing, placed in dishes containing droplets of fertilization medium, which were under mineral oil. Then, 1 × 10^6^ capacitated sperms from the sample of each mouse were added to oocytes in the droplets. Dishes were kept in an incubator at 37 C in 5% CO_2_. After 3–5 hr using an inverted microscope, the fertilization process was assessed by observing 2 pronuclei. Following the culture of the zygotes for 24 hr, the percentage for 2-cell embryos were calculated. The embryos were cultured for 120 hr, and finally, the percentages of blastocyst and arrested embryos were determined (19–21).

### Ethical Considerations

The study was performed according to the Guidelines for Care and Use of Laboratory Animals and was approved by the Ethics Committee of Urmia University of Medical Sciences, Urmia, Iran. (Code: IR.UMSU.REC.1398.103). To obtain enough oocytes with good quality for IVF analysis, we used 55 adult female mice that were approved by the Ethics Committee of Urmia University of Medical Sciences, Urmia, Iran.

### Statistical Analysis

The data obtained from the IVF method were analyzed using Minitab software (version 15.1; Minitab Inc., Boston, USA) using 2 proportional tests. Analysis of other data were done with Statistical Package for Social Sciences software (16; SPSS Inc., Chicago, USA) using one-way analysis of variance (ANOVA) accompanied by Tukey's post hoc test. All results were expressed as mean ± standard deviation, and differences were considered significant at p < 0.05.

## 3. Results

### Testicular histomorphometry

**Figure 1:**
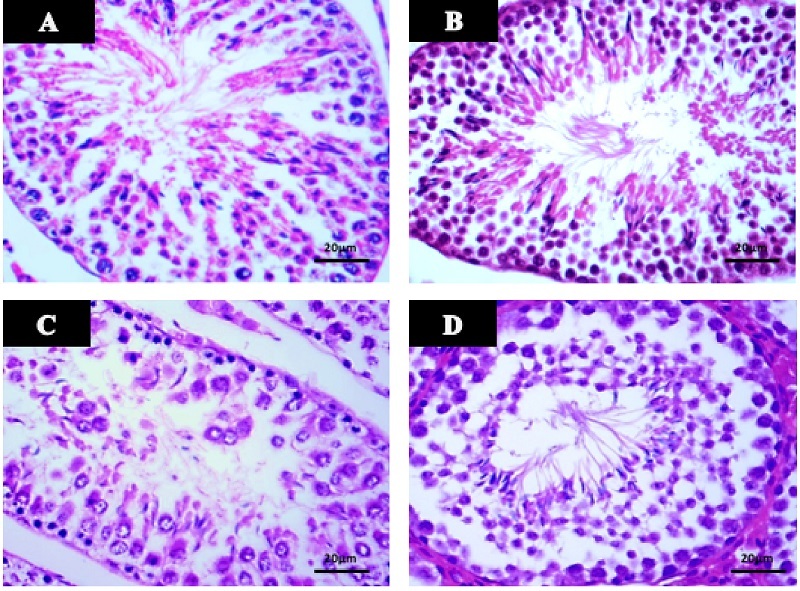
Histomorphological evaluations of the testicular tissues with hematoxylin and eosin staining. A) Control, B) MLT, C) AFB1, and D) AFB1 + MLT group. x400 magnification. Scale bar: 20 µm. MLT: Melatonin, AFB1: Aflatoxin B1.

Table I shows the effects of AFB1 and AFB1 + MLT on the SPI, diameter, and epithelial height of SNTs of male mice. No significant differences among the control and MLT groups were observed. Compared with the control, AFB1 treatment for 35 days caused a statistically significant reduction in the SPI, diameter, and epithelial height of SNTs (p < 0.001) (Figure 1). Administration of MLT + AFB1 caused a significant increase in these indexes compared to the AFB1 group (p < 0.027, p < 0.012, p < 0.036, respectively). However, a significant reduction was observed in the AFB1 + MLT group compared to the control group.

### Sperm parameters

**Figure 2:**
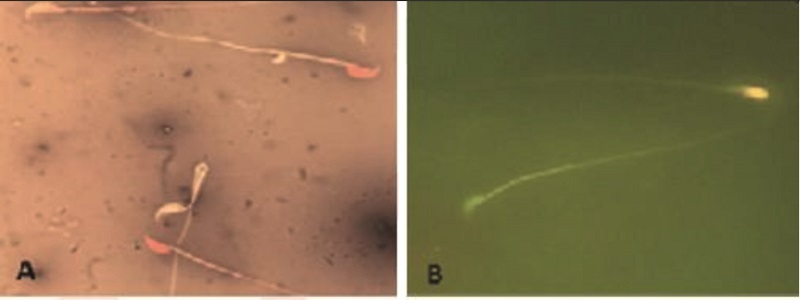
A) In the eosin-nigrosine staining test, alive unstained sperms indicated a white head and dead sperms a red head. B) Acridine orange staining test to detect sperm DNA damage, normal sperms are indicated with green color head and abnormal ones with yellow to red color.

The effects of AFB1 and AFB1 + MLT treatment on sperm parameter changes are presented in table II. No significant differences were observed between the control and MLT groups. Our data revealed that mice exposed to AFB1 showed a substantial decrease (p < 0.001) in count, motility, and viability of spermatozoa (Figure 2) compared to control and MLT groups. In contrast, these parameters in the group co-administrated by AFB1 + MLT were significantly higher relative to the AFB1 group (p < 0.001). Furthermore, as presented in table II, our results showed that mice in AFB1 group had a significant increase in sperm morphologic abnormalities compared to the control group (p < 0.001). Nevertheless, AFB1-exposed mice treated with MLT showed a dramatic decrease in abnormal spermatozoa compared to the AFB1 group (p < 0.001).

### Sperm chromatin condensation and DNA integrity

**Figure 3:**
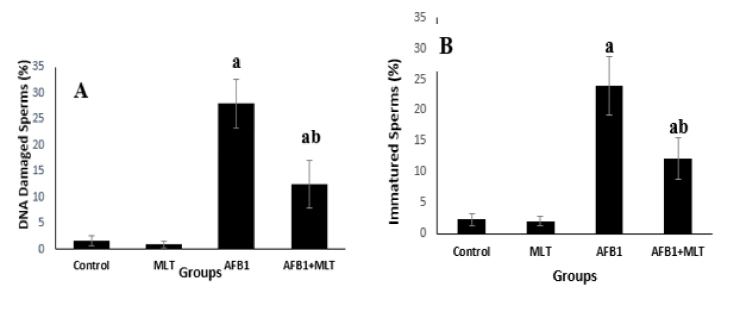
Effect of MLT on the percentages of sperm with A) DNA damage and B) Immature sperms in mice treated with AFB in different groups. a: Significant differences with control and MLT groups (p < 0.001), b: Significant differences with AFB1 group (p < 0.01). MLT: Melatonin, AFB1: Aflatoxin B1.

Aniline blue and acridine orange (Figure 1) staining tests showed that the percentages of immature sperms and DNA damage in sperms in mice exposed to AFB1 were significantly (p < 0.001) higher than control and MLT groups. It was indicated that the treatment of AFB1-exposed mice with MLT significantly (p < 0.01) decreased immature sperm and DNA damage compared to the AFB1 group (Figure 3).

### In vitro fertilization and embryo development

**Figure 4:**
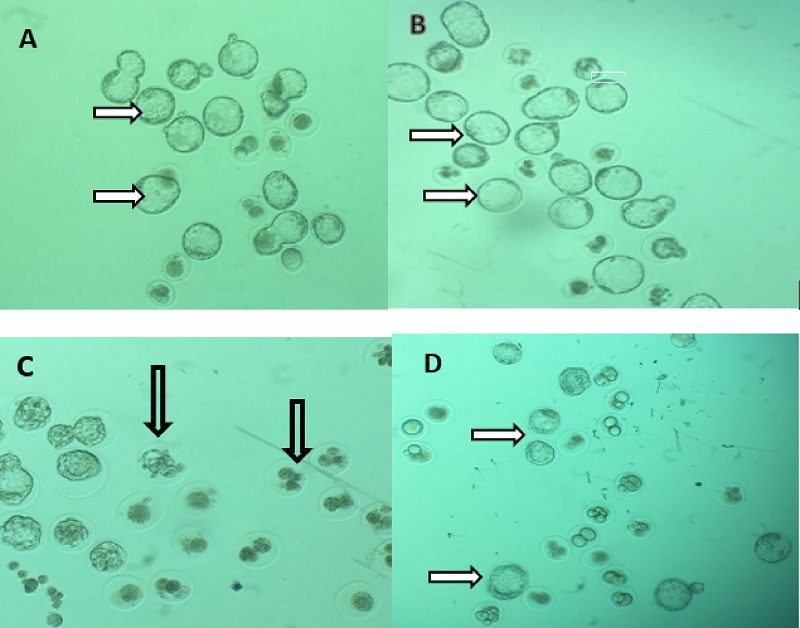
Representative images of the effects of AFB1 on A) Preimplantation embryos, and B) Control and MLT groups, respectively: Most of the embryos are at the blastocyst stage (thin arrows), C) AFB1 group: Most of the embryos are arrested and lysed (thick arrows), D) AFB1 + MLT group: Blastocyst formation (thin arrows) has been increased. AFB1: Aflatoxin B1, MLT: Melatonin.

All findings about IVF rate and embryo growth indifferent groups were presented in table III. Our results demonstrated that male mice exposed to AFB1 for 35 days decreased the percentages of fertilization, 2-cell and blastocysts embryos, and increased arrested embryos significantly (p < 0.001) compared to control group (Figure 4). Administration of MLT in AFB1-exposed mice elevated the rate of in vitro fertility as well as embryo development (2-cell and blastocysts formation) compared to AFB1 group (p < 0.001). In contrast, the percentage of arrested embryos significantly decreased relative to the AFB1 group (p < 0.036).

**Table 1:** The effects of MLT on SPI, tubular diameter, and epithelial height of SNTs of testicular tissue in mice treated with AFB1 in different groups

**Groups**	**Control**	**MLT**	**AFB1**	**AFB1 + MLT**	**P-value**
**SPI**	79.42 ± 3.59	77.14 ± 4.09	57.85 ± 4.14	64.85 ± 2.5	0.876^a^ 0.001^b^ 0.001^c^ 0.001^d^ 0.001^e^ 0.027^f^
**Tubular diameter (μm)**	220.17 ± 6.22	219.31 ± 6.69	174.86 ± 8.63	188.46 ± 8.03	0.995^a^ 0.001^b^ 0.001^c^ 0.001^d^ 0.001^e^ 0.012^f^
**Epithelial height (μm)**	71.89 ± 4.68	73.83 ± 7.03	54.83 ± 3.21	62.67 ± 4.42	0.935^a^ 0.001^b^ 0.019^c^ 0.001^d^ 0.005^e^ 0.036^f^
All data presented as Mean ± SD, One-way ANOVA test. a: Control vs. MLT, b: Control vs. AFB1, c: Control vs. MLT+AFB1, d: MLT vs. AFB1, e: MLT vs. MLT+AFB1, f: AFB1 vs. MLT+AFB1. MLT: Melatonin, SPI: Spermiogenesis index, SNT: Seminiferous tubules, AFB1: Aflatoxin B1					

**Table 2:** The effects of MLT on sperm parameters in mice treated with AFB1 in different groups

**Groups**	**Control**	**MLT**	**AFB1**	**AFB1 + MLT**	**P-value**
**Sperm count ( × 10^6^)**	78 ± 7.85	78 ± 9.83	45 ± 6.44	77 ± 12.02	0.997^a^ 0.001^b^ 0.969^c^ 0.001^d^ 0.599^e^ 0.001^f^
**Sperm motility (%)**	69.25 ± 2.98	66.5 ± 2.64	42.5 ± 6.24	64.66 ± 2.08	0.501^a^ 0.001^b^ 0.098^c^ 0.001^d^ 0.744^e^ 0.001^f^
**Sperm viability (%)**	77 ± 3.55	79.75 ± 3.5	47.5 ± 7.14	71 ± 2.88	0.718^a^ 0.001^b^ 0.099^c^ 0.001^d^ 0.011^e^ 0.001^f^
**Sperm morphologic abnormalities (%)**	8.75 ± 2.21	7.25 ± 2.06	36 ± 3.9	20 ± 3.6	0.886^a^ 0.001^b^ 0.001^c^ 0.001^d^ 0.001^e^ 0.001^f^
Data presented as Mean ± SD, One-way ANOVA test. a: Control vs. MLT, b: Control vs. AFB1, c: Control vs. MLT+AFB1, d: MLT vs. AFB1, e: MLT vs. MLT+AFB1, f: AFB1 vs. MLT+AFB1. MLT: Melatonin, AFB1: Aflatoxin B1					

**Table 3:** The effects of MLT on in vitro fertility and embryonic development in mice treated with AFB1 in different groups

**Groups**	**Control**	**MLT**	**AFB1**	**AFB1 + MLT**	**P-value**
**Number of oocytes**	195	170	287	242	–
**Fertilization rate (%)**	182 (93.32)	163 (95.88)	157 (56.47)	199 (82.23)	0.286^a^ 0.001^b^ 0.001^c^ 0.001^d^ 0.001^e^ 0.001^f^
**2-cell (%)**	160 (87.91)	152 (93.25)	127 (58.52)	144 (72.36)	0.066^a^ 0.001^b^ 0.001^c^ 0.001^d^ 0.001^e^ 0.001^f^
**Blastocyst (%)**	110 (60.43)	109 (66.87)	37 (17.05)	69 (34.67)	0.134^a^ 0.001^b^ 0.001^c^ 0.001^d^ 0.001^e^ 0.001^f^
**Arrested embryos (%)**	72 (39.56)	54 (33.12)	180 (82.94)	130 (65.32)	0.301^a^ 0.001^b^ 0.001^c^ 0.001^e^ 0.001^d^ 0.036^f^
Data are presented as n (%), 2 proportional tests. a: Control vs. MLT, b: Control vs. AFB1, c: Control vs. MLT+AFB1, d: MLT vs. AFB1, e: MLT vs. MLT+AFB1, f: AFB1 vs. MLT+AFB1. MLT: Melatonin, AFB1: Aflatoxin B1					

## 4. Discussion

Numerous environmental agents affect spermatogenesis and fertility. Among these factors, AFs especially AFB1, has been shown as a major risk factor for male infertility. In the current study, to evaluate the toxic effects of AFB1 and the possible protective role of MLT in reproductive functions of male mice, we evaluated the histopathological status of testis, sperm parameters quality, in vitro fertilization rate, and in vitro embryonic development. In the present study, the findings showed that AFB1 significantly decreased all sperm parameters and also induced damage to the testis and sperm DNA. Furthermore, IVF analysis revealed that administration of AFB1 in male adult mice reduced in vitro fertility and in vitro embryonic development. The application of MLT in these mice exposed to AFB1 attenuated the negative effects of AFB1. To our knowledge, this is the first study that confirms MLT supplementation efficiently restored the testicular function in AFB1 induced-testicular damage.

Sperm parameters such as density, motility, viability, and morphology are valid indices for evaluating testicular function and male fertility (7). The findings of our study indicated that mice exposed to AFB1 showed a remarkable reduction in sperm concentration and motility, which could be linked to impaired spermatogenesis status. In line with these findings, Huang et al. reported a significant decrease in sperm density and motility in mice treated with AFB1 (22). It was documented that decreased sperm motility could be associated with reduced testicular and epididymal proteins. The protein content in the epididymis is important for sperm maturation, motility, and fertility. Therefore, a decrease in protein synthesis due to AFB1 treatment might impair sperm function (23).

It has been revealed that oxidative stress is linked with a reduction of sperm quality, and many previous publications reported that AFB1 exposure leads to oxidative stress (6, 22, 24). However, we speculated that AFB1-induced testicular tissue damage and spermatogenesis disorder may result from oxidative stress. In the present study, the group of mice that received MLT along with AFB1 showed better spermatogenesis status with decreased testicular damage. These findings of our study may be due to the anti-apoptotic and antioxidant properties of MLT, as documented earlier. Although, in the current study, we did not evaluate apoptosis, we postulate that the anti-apoptotic action of MLT in testicular tissue could protect testis and spermatogenesis.

In agreement with our findings, an investigation in 2021 found that MLT improved the quality of all sperm parameters following busulfan treatment in mice (25).

In a previous study, it was reported that AFB1-induced testicular damage may result from abnormal autophagy which is positively associated with oxidative stress. Abnormal or excessive levels of autophagy following AFB1 treatment induces cell death and spermatogenesis disorder (6). However, one limitation of the present study is lack of stress oxidative evaluation which needs to be determined in future studies.

Previous studies have indicated that a decline in testosterone levels in mice exposed to AFB1 mice, are linked to spermatogenesis process impairment (3). In the present study, MLT treatment recovered AFB1-induced reduction of sperm motility and concentration, and it suggests that MLT may be effective in alleviating the reduction of testosterone levels and then could, at least partially, improve spermatogenesis. The expression of MLT receptors on mouse Leydig cells has been reported, and the knockdown of MLT receptors inhibited HCG-induced testosterone synthesis by inhibiting estrogen gene expression (26). However, it is assumed that the direct effect of MLT on Leydig cells modifies testosterone production which is essential for normal spermatogenesis.

In the current study, to further investigate the impact of AFB1 on spermatogenesis, sperm morphology and viability were also evaluated. Our findings indicated that the percentages of both sperms with normal morphology and viability were significantly reduced in the AFB1-treated group. A decrease in sperm viability and motility could be linked to mitochondrial dysfunction. It was revealed that mitochondrial damage is associated with reproductive toxicity and accompanied by infertility (27). Adverse effects of AFB1 on sperm mitochondria were also documented in, other previous studies. A study done in 2018 reported alterations in mitochondrial polarity in spermatozoa following AFB1 exposure (28). In line with these findings, a publication in 2018 evidenced that AFB1 significantly decreased the mitochondrial content of germ cells (5). Other researchers have attributed the abnormality of sperm morphology to sialic acid. Sialic acid as a sialoglycoprotein is important for maintaining the structural integrity of the sperm membrane and sperm maturation. Alteration of sialic acid in the testis and epididymis during aflatoxicosis indicated that AFB1, through inducing disintegrity of sperm membrane, might influence sperm morphology (23). Our findings showed that MLT application significantly decreased the abnormality of sperm morphology induced by AFB1 exposure. This finding is in accordance with a previous publication that reported that MLT significantly decreases the percentages of abnormal sperm morphology following nicotine treatment (16). However, further investigations are recommended to find out the effects of MLT on sialic acid in epididymis and testis.

DNA is known as a target molecule for toxic compounds. In the present study, we used acridine orange staining to evaluate sperm DNA damage. Our findings revealed that AFB1 treatment caused significant damage to sperm DNA. It has been shown that a high percentage of sperm with damaged DNA are associated with infertility (29).

Our findings demonstrated that the proportion of 2-cell embryos and blastocytes developed from AFB1-treated male mice were significantly lower than in the control group. In confirmation of our study, it was documented that the exposure of fresh semen to AFB1 leads to sperm DNA damage and impaired fertilization competence expressed by a declined percentage of oocytes that cleaved to 2- and 4-cell stages embryos (28). However, these results suggest that sperm DNA damage not only decreased the fertilization rate but also negatively affected embryonic development.

2 potential mechanisms for sperm DNA damage by AFB1 have been reported. The first one is direct interaction of AFB1 with the DNA, producing reversible noncovalent interaction. The second one is an indirect effect of the AFB1 metabolites. In the liver, AFB1 can be metabolized by cytochrome P450 enzymes into the genotoxic metabolite 8, 9-epoxide-AFB1 which binds to DNA and forms AFB1-DNA adducts, thus causing DNA fragmentation (30, 31). Previous studies have reported the binding of AFB1 to sperm DNA, which impairs normal spermatogenesis and eventually leads to a reduction of the fertilization competence (28, 32). As mentioned before, many in vitro and in vivo studies have shown that AFB1 can induce oxidative stress. Indeed, oxidative stress and genotoxic metabolites produced by AFB1 are the main reasons causing sperm DNA damage, leading to atypical spermatozoa and reducing male fertility (33).

In the current study, the fertilization rate and in vitro embryonic development of male mice exposed to AFB1 were significantly lower compared to control mice. The value of these parameters were restored nearly to the control level when AFB1 was co-administrated with MLT, but significant differences were still observed. These findings were supported by a previous study that reported that MLT improves in vitro fertilization and blastocyst formation rate in paclitaxel-induced mice spermatogenesis and fertility defects (34). The positive effects of MLT on testis histological changes and spermatogenesis indexes following treatment with dexamethasone and also the protective impacts of MLT on in vitro embryonic development in mice treated with doxorubicin were reported (35, 36). Meanwhile, the percentage of sperm with DNA damage had significantly decreased in the MLT + AFB1 group compared to the AFB1 group. In confirmation of our finding, it has been reported that MLT reduces sperm DNA fragmentation in nicotine-treated mice (16). It was also reported that the addition of MLT preserved DNA integrity in cryopreserved ram spermatozoa (37). It has been documented that oxidative stress is a major cause for DNA damage (38).

The exact mechanism of the protective effects of MLT on sperm DNA following AFB1 administration is not well elucidated. Given the inducing effects of aflatoxin in creating oxidative stress and the antioxidant properties of MLT, it is assumed that MLT through its antioxidant property may preserve the DNA. More studies are needed to explain it.

A significant rise in fertilization rate and embryonic growth after MLT administration may be linked to the protection of testicular tissue and the spermatogenesis process. In the present study, histopathological analysis of mice testes administrated with AFB1 for 35 consequent days showed various degenerative changes in SNTs and MLT administration restored them. The findings reported here are in accordance with previous publications reported that administration of AFB1 caused degraded changes in the testis, including atrophy of SNTs epithelium and sloughing of spermatogenic cells. AFB1-induced testicular damage may have resulted from abnormal autophagy, which is positively associated with oxidative stress (6). However, all AFB1-induced changes in testis were ameliorated by MLT administration.

## 5. Conclusion

AFB1 administration resulted in histopathological alterations in the testis and had adverse effects on all sperm parameters, percentage of invitro fertilization, and embryo development. These effects of AFB1 on male reproductive function are ameliorated by MLT supplementation, which might be due to its antioxidant and anti-apoptotic properties, shown previously. Our study provides the first evidence that the impaired IVF rate and in vitro embryonic development in male mice exposed to AFB1, improved by MLT treatment. The findings of the current study highlight the testicular toxicity of AFB1 and also suggest that MLT can be used as a promising agent against AFB1-induced damage to male reproductive organs and infertility.

##  Data Availability

Data supporting the findings of this study are available upon reasonable request from the corresponding author and first authors.

##  Author Contributions

M. Karimipour designed the study and had full access to all of the data in the study and takes responsibility for the integrity of the data and the accuracy of the data analysis. M. Sabahi: Acquisition, laboratory workup, and histological examination. A. Ahmadi and M. Sabahi: Acquisition, laboratory workup and analysis of sperm parameters and IVF data. M. Karimipour, Gh. Farjah and B. Pourheydar: Drafting of the manuscript. M. Karimipour and A. Ahmadi: Statistical analysis. M. Karimipour: Supervision and finalization of the article. Gh. Farjah and B. Pourheydar reviewed the article. All authors approved the final manuscript and take responsibility for the integrity of the data.

##  Conflict of Interest

The authors declare that they have no conflict of interest.
